# Tracking the Pulse of the Dunes: Seasonal Metabolic Responses of *Liolaemus arambarensis* to Climatic Variability

**DOI:** 10.1002/jez.70091

**Published:** 2026-04-27

**Authors:** Artur Antunes Navarro Valgas, Gustavo Kasper Cubas, Diogo Reis de Oliveira, Jessica Fonseca Araujo, Guendalina Turcato Oliveira, Laura Verrastro

**Affiliations:** ^1^ Herpetology Lab, Department of Zoology Universidade Federal do Rio Grande do Sul Porto Alegre Brazil; ^2^ Conservation Physiology Lab Pontifícia Universidade Católica do Rio Grande do Sul, School of Health and Life Sciences Porto Alegre Brazil; ^3^ Department of Physiology, Comparative Metabolism and Endocrinology Laboratory Universidade Federal do Rio Grande do Sul Porto Alegre Brazil

**Keywords:** biomarkers, conservation physiology, lizard, metabolism

## Abstract

Seasonal environmental fluctuations profoundly influence ectothermic vertebrates, regulating their physiology, metabolism, and life cycles. This study investigated the metabolic and morphometric seasonal dynamics of the subtropical sand lizard *Liolaemus arambarensis*, an endangered species endemic to the coastal dunes of southern Brazil. Over two annual cycles (July 2020 to May 2022), 133 individuals (43 males, 45 females, 45 juveniles) were sampled across all seasons. Through an ecophysiological and biochemical approach, we quantified intermediate metabolism biomarkers—including glucose, proteins, albumin, uric acid, lactate, triglycerides, cholesterol, glycogen, and total lipids—in plasma, liver, gonads, and skeletal muscles, alongside somatic and organ indices. Data were analyzed using generalized linear models (GLMs) with gamma distribution and log‐link, followed by type III ANOVA and Tukey post hoc tests. Marked sex‐ and age‐specific seasonal patterns were detected. Females exhibited strong reproductive investment in spring and summer, characterized by increased gonadal proteins and triglycerides, elevated plasma albumin and cholesterol, and depletion of hepatic and muscular reserves—consistent with vitellogenesis and egg production. Males initiated reproductive investment earlier, accumulating hepatic and muscular glycogen and lipids in autumn–winter and mobilizing these reserves during spring for spermatogenesis and reproductive behaviors. Juveniles displayed strategies oriented toward somatic growth and survival, with high tissue lipid content in summer (yolk‐derived reserves), hyperglycemia and hepatic glycogen accumulation in winter (suggesting cryoprotective or osmotic roles), and metabolic depletion in autumn, likely due to intraspecific competition for limited resources. The results reveal a plastic physiological strategy combining temperate‐like energy conservation during cold periods with tropical‐like reproductive allocation in warm months. Such metabolic flexibility underlies the species' adaptation to subtropical thermal variability and provides valuable predictive indicators for ecological modeling and conservation planning in the context of ongoing climate change.

## Summary

- First integrative ecophysiological study of *Liolaemus arambarensis*, an endangered sand lizard endemic to the subtropical dunes of southern Brazil, conducted over two full annual cycles (2020–2022).
- Comprehensive analysis of intermediate metabolism biomarkers (proteins, lipids, carbohydrates, and energy reserves) across multiple tissues—plasma, liver, gonads, and muscles—revealing seasonal physiological adjustments linked to reproduction, growth, and maintenance.
- Females exhibited marked seasonal metabolic shifts, with reproductive investment peaking in spring and summer, characterized by mobilization of hepatic and muscular reserves for vitellogenesis and egg production.
- Males showed early gonadal maturation beginning in autumn–winter, accumulating glycogen and lipids to support spermatogenesis and reproductive behaviors in spring.
- Juveniles displayed ontogeny‐specific metabolic strategies, relying on yolk‐derived lipids in summer, maintaining high glucose and glycogen in winter (suggesting cryoprotective adjustments), and showing metabolic depletion in autumn due to competition for resources.
- The study demonstrates a physiological plasticity that integrates temperate‐like overwintering strategies with tropical reproductive cyclicity, reflecting adaptive flexibility to subtropical thermal variability.
- Findings provide novel biomarkers and seasonal metabolic profiles that can be applied to predictive models of reptile responses to environmental change, supporting conservation and management efforts for Neotropical lizards under climate stress.

## 1 Introduction

The cyclic fluctuations in environmental variables generated by seasonality govern the life cycle of organisms on Earth, regulating their reproduction, growth, and physiology (Lincoln 2019; Capdevila et al. 2022). Seasons are marked by predicted variations in abiotic variables such as temperature, luminosity, salinity, and oxygen levels, which play fundamental roles in determining organisms' biochemical and behavioral responses, leading to physiological acclimatization, reproductive signaling and regulation, and altered behavior and activity patterns (Longhini et al. 2019; Oliveira et al. 2021). Non‐avian reptiles, as ectothermic organisms, are greatly influenced by abiotic factors, in a manner such that solar radiation, temperature, water cycle, and photoperiod regulate major life cycle changes, dictating periods of high and low metabolic activity (Naya et al. 2008; Liz et al. 2019).

Although numerous studies have demonstrated physiological responses to climatic seasonality in lizards, including species from the Liolaemidae family, subtropical reptiles remain comparatively underrepresented in the literature. For instance, *Liolaemus tandiliensis* has been shown to exhibit marked seasonal shifts in thermal biology, adjusting its thermoregulatory behavior and physiological traits across the year (Stellatelli et al. 2018). *Liolaemus parvus* and *Phymaturus extrilidus*, two syntopic species from the Puna region of Argentina, demonstrate distinct thermal strategies that reflect seasonal and microhabitat variation (Gómez Alés et al. 2017). Furthermore, a recent meta‐analysis by Giacometti et al. (2024) highlighted how seasonality influences thermal biology across lizard species with varying thermoregulatory strategies, yet also pointed out the geographic bias toward temperate‐zone studies. Field‐based studies, especially involving endemic and endangered species, are essential for developing predictive models and informing conservation strategies (Gunderson and Stillman 2014; Pereira 2021).

The use of physiological biomarkers is crucial for understanding organisms' adaptive dynamics in response to annual and seasonal environmental variations (Wikelski and Cooke 2006). They also help predict and monitor species' adaptive fitness in response to climate changes caused by human impact (Domenici and Seebacher 2020; A. Valgas et al. 2024; Araujo et al. 2024). In particular, biomarkers of intermediate metabolism provide valuable insights into an animal's health status and can serve as proxies for fitness, reflecting organismal energy budget reserves and mobilized metabolites that ultimately sustain and ensure reproduction success, growth, development, and activity patterns (Beaulieu and Costantini 2014; Hau et al. 2015). In this context, the overlooked and poorly understood ecophysiological dynamics of subtropical organisms represent a significant gap in the literature and may hinder our understanding of how these species will cope with climate change in naturally variable environments (Liz et al. 2019; Dayananda et al. 2021; Cox et al. 2022; Li et al. 2025).

Given the current scenario of global climate change, understanding the physiological mechanisms that enable ectothermic organisms to survive in naturally variable environments has become increasingly urgent. Seasonal physiology, particularly regarding thermoregulation and energy metabolism, offers valuable insights into how these animals cope with environmental stressors (A. Valgas et al. 2024). However, most research in this field focuses mainly on the thermal biology of species from tropical and temperate regions, leaving critical knowledge gaps concerning the physiological/biochemical responses of subtropical reptiles (Mi et al. 2022; Giacometti et al. 2024).

In this context, *Liolaemus arambarensis*, a sand lizard species restricted to the coastal dunes of Rio Grande do Sul, southern Brazil (Verrastro et al. 2003; Cubas et al. 2024), represents a promising model for investigating seasonal metabolic adaptations in a subtropical sandy habitat (Verrastro et al. 2003; A. Valgas et al. 2024). According to the IUCN, the species is currently classified as Endangered (Silveira et al. 2021), primarily due to habitat loss, fragmentation, and its marked microendemism (Cubas et al. 2024; IUCN 2021). *L. arambarensis* is a diurnal, omnivorous, and heliothermic species (Verrastro et al. 2003; Liz et al. 2019), exhibiting a well‐established seasonal reproductive cycle and activity pattern, with activity concentrated between September and March. Within this framework, the present study aims to investigate, through an ecophysiological and biochemical approach, variations in metabolic and morphometric parameters of males, females, and juveniles throughout the intra‐annual cycle. By quantifying biomarkers of intermediate metabolism and energy reserves across different tissues, we seek to elucidate how this species allocates metabolic resources in response to seasonal shifts and physiological demands. We hypothesize that *L. arambarensis* exhibits a marked seasonal cycle, with dietary energy intake allocated to tissue reserves during autumn and winter, leading to the formation of fat bodies, as previously observed by Verrastro (2001). During the warmer seasons (spring and summer), females are expected to mobilize tissue and fat body reserves—particularly proteins and triglycerides—to enhance egg production and vitellogenin synthesis, while carbohydrates are likely used to sustain agonistic and reproductive behaviors. In contrast, males are expected to rely primarily on carbohydrate and triglyceride reserves to support gamete production and reproductive activities, resulting in the depletion of fat bodies during this period (Verrastro 2001). Collectively, these findings are expected to provide novel insights into the physiological ecology and natural history of Neotropical reptiles, contributing to a broader understanding of adaptive and plastic responses of subtropical lizards under changing environmental conditions.

## 2 Materials and Methods

### 2.1 Licenses and Ethics Committee

**Figure 1:**
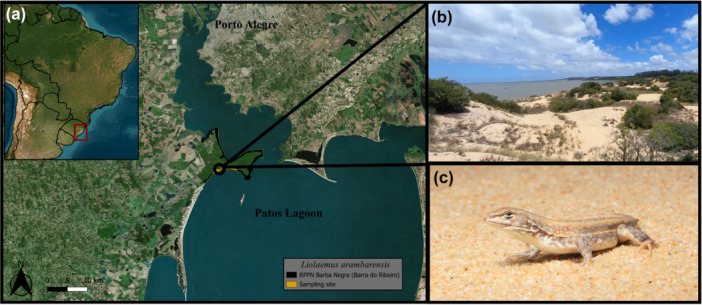
Geographic location and habitat of *Liolaemus arambarensis*. (a) Map showing the sampling site (yellow area) within the RPPN Barba Negra (Barra do Ribeiro, Rio Grande do Sul, Brazil), on the western margin of the Patos Lagoon, approximately 60 km south of Porto Alegre. (b) Typical coastal dune habitat where the species occurs, characterized by sandy soils and sparse vegetation dominated by shrubs and grasses. (c) Adult *L. arambarensis* specimen photographed in situ on the sand substrate, illustrating its cryptic coloration adapted to the open dune environment.

This project obtained the proper license for collection, transport, and use of biological material from the Chico Mendes Institute for Biodiversity Conservation (ICMBio) (SISBIO No. 75139‐1) and authorization from the Ethics Committee for Animal Use of the Federal University of Rio Grande do Sul (CEUA‐UFRGS No. 38872). A total of 133 individuals of *L. arambarensis* (43 males, 45 females, and 45 juveniles), adult individuals were considered as those with minimum snout‐vent length (SVL) of 42 mm for females and 45 mm for males, following the species description (Verrastro et al. 2003), were collected in the municipality of Barra do Ribeiro, RS, Brazil (30°24′43″ S 51°13′03″ W) over two seasonal cycles (July 2020 to May 2022) (Figure 1). Seasonal sampling included 10−13 individuals per group in each season, totaling: winter: 11 males, 10 females, and 12 juveniles; in spring: 10 males, 12 females, and 11 juveniles; summer: 12 males, 13 females, and 10 juveniles; and autumn: 10 males, 10 females, and 12 juveniles. Adults of *L*. *arambarensis* exhibit clear sexual dimorphism, facilitating sex identification during handling. Females possess two to four genital pores and white ventral scales, whereas males have four to six genital pores and may display orange lateral scales interspersed with blue, as well as red‐tipped lips (secondary sexual characteristics). Juveniles were identified based on SVL, with individuals measuring less than 42 mm classified as juveniles (Verrastro et al. 2003).

### 2.2 Collection and Sampling

Individuals were collected by hand during active searches, between 09:00 and 16:00. Immediately after capture, the animals were euthanized with Ketamine 10% (dextroketamine hydrochloride), which included a 2 mg dose injected close to the heart. SVL and total length (TL) were measured with a 0.1 mm precision caliper. Body mass was determined with a semi‐analytical balance with 0.01 g precision. The specimens were then dissected to remove liver, gonads (ovaries and testicles), and caudal and thigh skeletal muscles.

Tissues were weighed on an analytical balance with 0.0001 g precision. All organs and blood samples were aliquoted, weighed, and stored in a −80°C freezer for further analysis. The biochemical analyses of tissue metabolites and oxidative balance were conducted using spectrophotometry. After the laboratory procedures for biological material extraction, the animals were deposited in the Scientific Collection of the Herpetology Laboratory at UFRGS (Nos. 7815 and 7816). The procedures are classified by the UFRGS Animal Ethics Committee (CEUA) as Non‐recovery, meaning “Procedures conducted under general anesthesia from which the animal does not regain consciousness,” according to CONCEA (2013).

### 2.3 Tissue Indices

The gonadosomatic index (IG) was determined as follows:

$$\text{IG}=\left(\frac{\text{Mg}}{\text{Mt}}\right)\times 100$$

Where Mg is the gonadal mass, and Mt is the total body mass of the individual.

The hepatosomatic index (IH) was determined as follows:

$$IH=\left(\frac{Mh}{Mt}\right)\times 10$$

Where Mh is the liver mass, and Mt is the total body mass of the individual.

The body fat index was determined as follows:

$$IGC=\left(\frac{Mcg}{Mt}\right)\times 100$$

Where Mcg is the abdominal fat body mass, and Mt is the total body mass of the individual.

The caudal myosomatic index was determined as follows:

$$IMCA=\left(\frac{Mma}{Mt}\right)\times 100$$

Where Mma is the caudal muscle mass, and Mt is the total body mass of the individual.

The thigh myosomatic index was determined as follows:

$$IMCO=\left(\frac{Mna}{Mt}\right)\times 100$$

Where Mmo is the thigh muscle mass, and Mt is the total body mass of the individual.

### 2.4 Degree of Stomach Fullness

Stomachs were categorized into classes based on the degree of stomach fullness (GR), visually determined according to a six‐class scale (Valgas et al. 2020):

Class 1 = 0%−empty;

Class 2 = < 5%−partially empty;

Class 3 = 5%−35%−empty to half full;

Class 4 = 35%−65%−half full;

Class 5 = 65%−95%−half full to full;

Class 6 = > 95%−full.

### 2.5 Intermediate Metabolism—Plasma

The levels of glucose, total proteins, albumin, uric acid, lactate, triglycerides, and total cholesterol were quantified in the blood using commercial colorimetric spectrophotometry kits from *Biotécnica: Biotecnologia Avançada*.

### 2.6 Tissue

Glycogen was extracted according to Van Handel (1965) and quantified using a commercial colorimetric glucose oxidase spectrophotometry kit from *Biotécnica: Biotecnologia Avançada*. Total proteins were quantified using a colorimetric spectrophotometry kit from *Biotécnica: Biotecnologia Avançada* from a phosphate buffer (20 mM), potassium chloride (140 mM), and protease inhibitor (1 mM) homogenization medium in the proportion of 6 mL:1 mg of tissue. Total lipids were extracted using the method of Folch et al. (1957) and measured using the sulfo‐phospho‐vanillin method (Frings and Dunn 1970). Triglycerides were extracted using the method of Folch et al. (1957) and quantified using specific kits from *BioTécnica: Biotecnologia Avançada*.

### 2.7 Statistical Analysis

The effects of group (sex and age), season, and their interaction on biochemical and morphometric markers were tested using generalized linear models (GLMs). Data were modeled assuming a Gamma distribution, with a log link function. ANOVA type III analysis of deviance tables was used to evaluate the significance of main effects and their interaction. When significant effects were detected (*p* ≤ 0.05), post hoc pairwise comparisons were performed using Tukey's adjustment for multiple testing. All analyses were conducted in R version 4.5.1 (R Core Team 2025), using the packages car. For type III ANOVA and emmeans. For estimated marginal means and post hoc contrasts.

## 3 Results

### 3.1 Morphometric and Feeding Indices

GLM revealed significant effects of group (females, males, and juveniles), season, and their interaction on most morphometric parameters. Body mass was consistently higher in males across all seasons, whereas juveniles exhibited the lowest values. A clear seasonal pattern was detected, with higher body mass during winter and a decline in autumn. The ventral volume index (VVI) followed a similar trend, being greater in males and peaking in winter, with reduced values in summer and autumn.

Reproductive investment, assessed through the IG, differed markedly among groups and seasons. Males exhibited higher IG values than females and juveniles overall, particularly during colder months. Females, however, showed a pronounced increase in IG during spring and summer, indicating active reproductive investment, whereas juveniles maintained consistently low values throughout the year. The IH also varied significantly, with females displaying higher values in autumn, suggesting seasonal shifts in energy allocation.

Muscle indices showed contrasting patterns. Juveniles exhibited lower branchial muscle mass index (MMI), with no strong seasonal variation. In contrast, the pectoral muscle mass index (PMI) was significantly higher in juveniles, particularly during summer and autumn, indicating differential somatic investment during growth (Table 1).

**Table 1:** Seasonal contrast in morphometric biomarkers.

	Winter	Spring	Summer	Autumn
Mass (g)				
Female	2.1 ± 0.4A	1 ± 0.36A	2.07 ± 0.23A	1.9 ± 0.27A
Male	3.27 ± 0.61A	2.8 ± 0.51A	3.08 ± 0.22A	2.5 ± 0.42A
Juvenile	1.02 ± 0.21A	0.52 ± 0.09A	0.95 ± 0.19A	0.74 ± 0.15A
CRC (mm)				
Female	44.21 ± 0.52A	46.52 ± 1.27A	48.1 ± 0.75A	45.58 ± 1.28A
Male	49.83 ± 1.77A	55.57 ± 1.92B	51.88 ± 1.19B	48.91 ± 1.76A
Juvenile	35.94 ± 0.9AB	39.8 ± 1.19B	31.64 ± 1.84C	33.22 ± 1.53AC
IG (%)				
Female	0.78 ± 0.23A	5.22 ± 1.51B	5.04 ± 1.48B	0.31 ± 0.06A
Male	1.91 ± 0.35AB	2.09 ± 0.27B	0.52 ± 0.11C	1.25 ± 0.26A
IH (%)				
Female	3.28 ± 0.58A	7.57 ± 1.04B	3.88 ± 0.47A	4.18 ± 0.53A
Male	2.53 ± 0.39A	3.39 ± 0.59A	2.00 ± 0.15A	2.74 ± 0.38A
Juvenile	4.97 ± 1.09A	8.81 ± 1.71B	1.7 ± 0.22A	2.38 ± 0.53A
IGC (%)				
Female	1.14 ± 0.38A	0.21 ± 0.14B	0.0052 ± 0.0002B	2.71 ± 0.48A
Male	0.76 ± 0.32A	0.22 ± 0.11A	0.55 ± 0.19A	0.82 ± 0.45A
IMA (%)				
Female	11.24 ± 3.19A	17.83 ± 2.99B	7.88 ± 3.63A	13.17 ± 2.15AB
Male	13.22 ± 2.88A	13.01 ± 1.54A	9.27 ± 0.58A	15.08 ± 1.62A
Juvenile	22.95 ± 6.15A	24.05 ± 3.23A	7.1 ± 0.29B	9.37 ± 1.26B
IMO (%)				
Female	9.72 ± 1.79A	22.91 ± 3.97B	9.1 ± 1.01A	10.6 ± 1.36A
Male	10.05 ± 1.61A	12.4 ± 1.53A	8.39 ± 0.41A	10.58 ± 1.31A
Juvenile	17.93 ± 3.89A	40.99 ± 8.92B	3.83 ± 0.45A	18.44 ± 6.28B

*Note:* Values are presented as the mean and standard deviation of each biomarker across seasons. The seasonal contrast was statistically analyzed using generalized linear models (GLMs) with an *⍺* level of 0.05. Statistical significance was further explored via post hoc pairwise comparisons with Tukey's adjustment for multiple testing. Within the table, lowercase letters indicate statistically significant differences between different groups within the same season, while uppercase letters denote significant differences among the distinct seasons within the same group.

Stomach fullness varied across groups and seasons, reflecting differences in feeding activity. Females exhibited lower stomach fullness during winter, moderate values in spring, and high fullness during summer, followed by a reduction in autumn. Males showed a more heterogeneous pattern, with bimodal distributions in winter and high stomach fullness during spring and summer. Juveniles displayed moderate stomach fullness in winter, increased feeding in spring and summer, and intermediate values in autumn (Table 2).

**Table 2:** Degree of stomach repletion in males, females, and juveniles of *Liolaemus arambarensis* throughout the seasonal cycle.

Season	Repletion degree (empty to full)
(A) Female	
Winter	1%–40%, 2%–10%, 3%–10%, 4%–10%, 5%–30%
Spring	1%–36%, 2%–10%, 3%–18%, 4%–9%, 5%–27%
Summer	1%–31%, 2%–8%, 3%–23%, 4%–10%, 5%–38%
Autumn	1%–40%, 2%–10%, 3%–10%, 4%–20%, 5%–20%
(B) Male	
Winter	1%–30%, 2%–30%, 3%–20%, 4%–20%
Spring	1%–60%, 2%–40%
Summer	1%–8%, 2%–8%, 3%–17%, 4%–17%, 5%–50%
Autumn	1%–30%, 2%–20%, 3%–50%
(C) Juvenile	
Winter	1%–45%, 2%–18%, 3%–9%, 4%–9%
Spring	1%–36%, 2%–9%, 3%–9%
Summer	1%–30%, 2%–10%, 3%–10%, 4%–20%
Autumn	1%–33%, 2%–25%, 3%–17%, 4%–17%

*Note:* (A) Stomach repletion in female *L. arambarensis*; (B) Stomach repletion in male *L. arambarensis*; (C) Stomach repletion in juveniles of *L. arambarensis*. Categories 1, 2, and 3 were considered as empty to medium stomachs in terms of food content, while categories 4, 5, and 6 were considered as medium to full stomachs in terms of food content.

### 3.2 Gonadal Biochemistry

**Figure 2:**
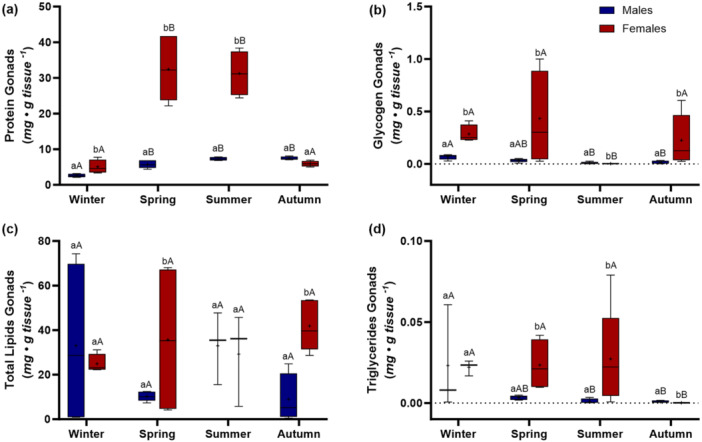
Seasonal variation in gonadal biochemical composition of males (blue) and females (red). (a) Protein, (b) glycogen, (c) total lipids, and (d) triglycerides (mg·g⁻¹ tissue). Values are presented as boxplots (median, 25–75 interquartile range). Different lowercase letters indicate significant differences among seasons within the same sex, while uppercase letters denote significant differences between sexes within the same season (*p* < 0.05).

The biochemical composition of gonads showed strong sex‐ and season‐dependent variation (Figure 2). Gonadal protein content (PT) was significantly influenced by sex, season, and their interaction. Females consistently exhibited higher protein concentrations than males, with pronounced seasonal variation characterized by marked peaks in spring and summer. These values were approximately sixfold higher than those observed in winter and autumn, indicating intense reproductive investment. In contrast, males maintained low and relatively stable protein concentrations throughout the year. Glycogen content followed a similar pattern, with females exhibiting higher concentrations than males in most seasons, particularly in spring. In both sexes, gonadal glycogen declined sharply during summer, reaching minimal values. Total lipid content showed no main effects of sex or season; however, a significant interaction revealed that females accumulated higher lipid concentrations in spring and autumn. Triglyceride levels also showed a significant sex‐by‐season interaction, with females showing higher concentrations in spring and summer, whereas males maintained consistently low levels.

### 3.3 Skeletal Muscle Biochemistry

**Figure 3:**
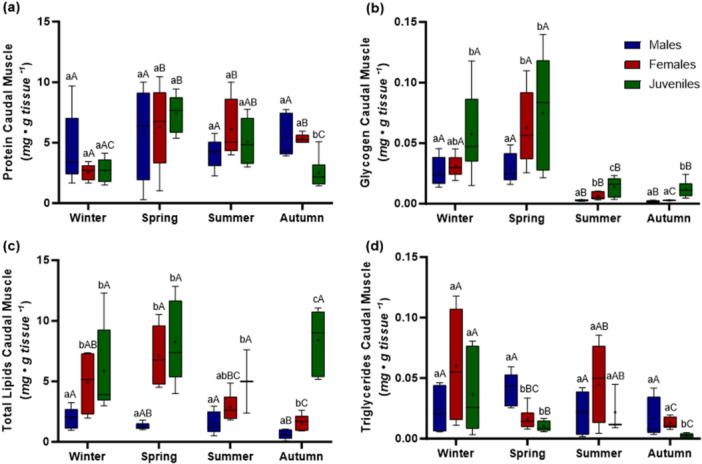
Seasonal variation in caudal muscle biochemical composition of males (blue), females (red), and juveniles (green). (a) Protein, (b) glycogen, (c) total lipids, and (d) triglycerides (mg·g⁻¹ tissue). Values are shown as boxplots (median, 25–75 interquartile range). Different lowercase letters indicate significant differences among seasons within the same group, whereas uppercase letters denote significant differences among groups within the same season (*p* < 0.05).

In the caudal musculature (Figure 3), PT varied according to group and season. Juveniles exhibited significantly lower protein concentrations in autumn compared to adults. Females and juveniles showed similar seasonal patterns, with higher protein levels in spring and summer and reduced values in winter and autumn, whereas males did not display significant seasonal variation. Glycogen concentrations were consistently higher in juveniles across all seasons and exhibited marked seasonal fluctuations in all groups, peaking in winter and spring and declining sharply in summer and autumn. Total lipid content differed significantly among groups, with females and juveniles presenting substantially higher levels than males throughout the year. While males and females showed seasonal variation, juveniles maintained relatively stable lipid levels. Triglyceride concentrations were primarily influenced by seasonal variation, with males exhibiting higher values than females and juveniles during spring and autumn.

**Figure 4:**
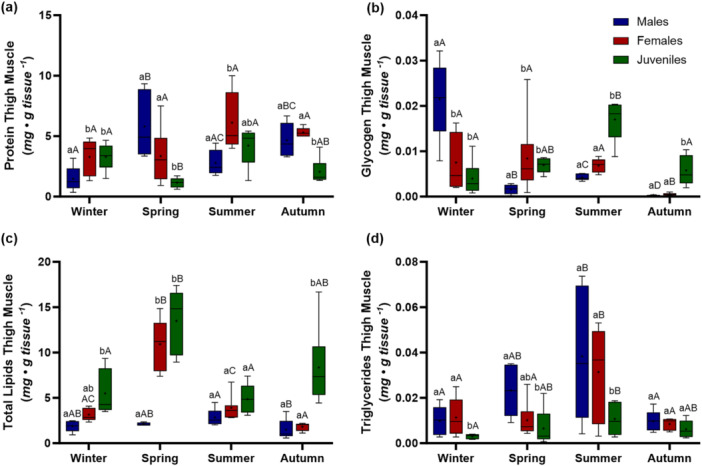
Seasonal variation in thigh muscle biochemical composition of males (blue), females (red), and juveniles (green). (a) Protein, (b) glycogen, (c) total lipids, and (d) triglycerides (mg·g⁻¹ tissue). Values are presented as boxplots (median, 25–75 interquartile range). Different lowercase letters indicate significant differences among seasons within the same group, whereas uppercase letters denote significant differences among groups within the same season (*p* < 0.05).

Thigh muscle biochemistry (Figure 4) revealed pronounced group‐ and season‐specific patterns. Protein concentrations were consistently lower in juveniles compared to adults, particularly during spring, summer, and autumn. Seasonal variation differed among groups: males exhibited peak protein levels in spring and autumn, juveniles in winter and summer, while females showed relatively stable values. Glycogen levels were higher in juveniles than in adults in most seasons, except in winter, when males exhibited peak concentrations. Adults showed a progressive decline in glycogen toward autumn, whereas juveniles peaked in summer. Total lipid content was markedly higher in juveniles across all seasons, while females showed pronounced seasonal variation, peaking in spring. Males maintained low and relatively stable lipid concentrations. Triglyceride levels were higher in adults than in juveniles and showed a clear seasonal pattern, with maxima in summer and minima in autumn and winter.

### 3.4 Hepatic Biochemistry

**Figure 5:**
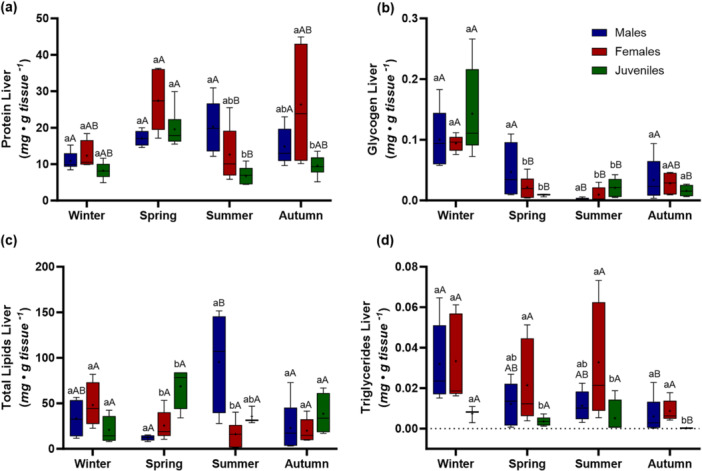
Seasonal variation in liver biochemical composition of males (blue), females (red), and juveniles (green). (a) Protein, (b) glycogen, (c) total lipids, and (d) triglycerides (mg·g⁻¹ tissue). Values are presented as boxplots (median, 25–75 interquartile range). Different lowercase letters indicate significant differences among seasons within the same group, whereas uppercase letters denote significant differences among groups within the same season (*p* < 0.05).

Liver PT (Figure 5) varied significantly among groups and seasons, with juveniles exhibiting markedly lower concentrations than adults during summer and autumn. All groups displayed peak protein levels in spring, followed by stabilization or decline in subsequent seasons. Hepatic glycogen showed strong seasonal variation, with maximum values in winter across all groups and a sharp decline during spring and summer. Group differences were detected primarily in spring and summer, reflecting sex‐ and age‐specific metabolic strategies. Total lipid content in the liver was strongly influenced by the interaction between group and season. Juveniles and females accumulated higher lipid concentrations in spring, whereas males showed pronounced lipid accumulation during summer. Hepatic triglycerides exhibited minor group differences and followed a similar seasonal trend in all groups, with higher values in winter and minimal concentrations in autumn.

### 3.5 Plasma Biochemistry

Plasma metabolites and proteins displayed both group‐ and season‐specific variation (Table 3). Glucose concentrations differed between adults and juveniles in spring and autumn, with adults exhibiting higher values. Females and juveniles showed peak glycemia in winter, followed by a seasonal decline, whereas males maintained stable levels year‐round. Plasma protein concentrations were higher in adults than juveniles during summer and autumn. Seasonal patterns differed among groups: males peaked in summer, females showed minimal winter values, and juveniles peaked in spring. Albumin levels were generally higher in juveniles than adults, except in summer, and showed seasonal peaks during warmer months in adults. Uric acid varied seasonally in females and juveniles, with higher values in winter and spring, whereas males showed no significant variation. Lactate levels remained stable across groups and seasons. Plasma triglycerides were influenced exclusively by season, with the highest concentrations in winter and progressive declines toward autumn. Total cholesterol differed markedly between adults and juveniles in summer and autumn, with adults showing substantially higher values. Seasonally, adults peaked in spring and summer, while juveniles exhibited a sharp spring peak followed by low concentrations in subsequent seasons.

**Table 3:** Seasonal contrast in plasma metabolic parameters using generalized linear models (GLMs, *⍺* 0.05).

	Winter	Spring	Summer	Autumn
Glucose (mg/dL)				
Female	33.2 ± 5.22A	27.7 ± 3.46A	18.41 ± 2.59B	27.67 ± 5.85A
Male	30.72 ± 5.43A	29.49 ± 3.38A	27.25 ± 3.11A	26.79 ± 4.63A
Juvenile	27.98 ± 4.39A	16.32 ± 1.86AB	14.09 ± 3.76AB	9.99 ± 1.87B
Total protein (g/dL)				
Female	0.17 ± 0.02A	0.27 ± 0.04A	0.30 ± 0.03A	0.30 ± 0.06A
Male	0.12 ± 0.03A	0.33 ± 0.05AB	0.47 ± 0.07B	0.37 ± 0.02B
Juvenile	0.19 ± 0.03AB	0.36 ± 0.02A	0.20 ± 0.03AB	0.09 ± 0.02B
Albumin (g/dL)				
Female	0.03 ± 0.008A	0.20 ± 0.03B	0.17 ± 0.006B	0.03 ± 0.009A
Male	0.03 ± 0.003A	0.13 ± 0.015BC	0.23 ± 0.015B	0.06 ± 0.014AC
Juvenile	0.11 ± 0.021AC	0.27 ± 0.008B	0.15 ± 0.005AB	0.071 ± 0.006C
Uric acid (mg/dL)				
Female	0.76 ± 0.09AB	1.09 ± 0.15A	0.58 ± 0.06B	0.56 ± 0.11B
Male	0.93 ± 0.19A	1.28 ± 0.17A	0.77 ± 0.09B	0.77 ± 0.11A
Juvenile	1.03 ± 0.09A	0.85 ± 0.15AB	0.43 ± 0.07B	0.46 ± 0.03B
Lactate (mg/dL)				
Female	3.13 ± 0.97A	12.22 ± 2.0B	10.79 ± 1.78B	9.06 ± 1.15AB
Male	9.80 ± 1.99A	7.42 ± 1.41A	13.81 ± 1.91A	9.77 ± 1.36A
Juvenile	9.48 ± 1.73A	8.29 ± 1.62A	8.75 ± 2.09A	6.51 ± 2.02A
Triglycerides (g/dL)				
Female	48.74 ± 4.91A	30.87 ± 5.4A	28.5 ± 5.84AB	11.69 ± 2.55B
Male	50.17 ± 2.76A	33.11 ± 4.93AB	24.8 ± 6.49BC	11.36 ± 1.99C
Juvenile	25.79 ± 4.12A	21.3 ± 1.14B	14.61 ± 2.53AB	11.38 ± 2.17B
Total cholesterol (mg/dL)				
Female	5.77 ± 0.87A	87.0 ± 1.99B	88.79 ± 0.88B	7.08 ± 1.53A
Male	4.09 ± 0.56A	82.33 ± 1.38B	88.93 ± 1.08B	6.76 ± 1.56A
Juvenile	6.15 ± 1.17A	86.38 ± 0.78B	5.07 ± 0.51A	3.16 ± 0.24A

*Note:* Values are presented as the mean and standard deviation. Lowercase letters indicate statistically significant differences between groups within the same season, while uppercase letters denote significant differences among seasons within the same group, as determined by post hoc pairwise comparisons with Tukey's adjustment for multiple testing.

## 4 Discussion

This is the first study to demonstrate the seasonal biochemical and functional adjustments exhibited by *L. arambarensis* throughout its life cycle. The information gathered in this study provides important evidences into the ecological dynamics of the species' energy budget, highlighting the numerous and distinct cycles of energy intake, storage, and expendure/allocation employed by adults and juveniles in response to environmental change. The analysis of intermediate metabolism biomarkers provided a deeper understanding into the main metabolites and byproducts associated with periods of reproductive engagement (e.g., agonistic behaviors, gamete production, and copulation), growth, development, and overall maintenance in *L. arambarensis*. Several studies have emphasized the importance of ecophysiological assays in natural environments for predicting species' adaptive fitness in response to environmental stressors, as these biomarkers directly indicate the health status and physiological condition of the animal, providing a good indicator of population health and fitness (A. Valgas et al. 2024; Li et al. 2025; Ling and Bonebrake 2022; Y. Wang et al. 2022; Costantini 2022).

As demonstrated by Verrastro et al. (2003), no statistically significant seasonal variation was detected in female SVL (*p *> 0.05), likely reflecting age homogeneity among the adult individuals analyzed (Table 1). Similarly, body mass did not differ significantly across seasons, although a slight reduction in Spring may be associated with reproductive activity, such as abdominal fat mobilization and clutch deposition (Verrastro 2001). The absence of significant variation in these somatic parameters does not preclude underlying metabolic and physiological adjustments, as body mass alone is an imprecise indicator of condition. Seasonal reallocations of energy among fat bodies, reproductive tissues, organ function, and maintenance have been documented in reptiles without corresponding changes in total mass (Wikelski and Thom 2000; Angilletta and Sears 2001; LeBas and Marshall 2000), and hepatic glycogen storage may further obscure energetic shifts due to its association with water retention (Clavijo‐Baquet et al. 2022). In this context, the lack of seasonal variation in *L. arambarensis* body mass underscores the importance of biochemical and physiological indices. Notably, during Winter, individuals exhibited the lowest IG and highest ICG values (*p* < 0.05; Table 1), consistent with increased energetic allocation to maintenance under cold conditions and reduced food availability, as corroborated by the high proportion of empty or partially empty stomachs (70%; categories 1–3, Table 2) (Verrastro 2001; Holden et al. 2021), indicating a seasonal reduction in reproductive investment in favor of survival.

Consistent with the absence of seasonal variation in somatic traits and the inferred reallocation of energy toward maintenance during Winter, metabolic patterns further support this interpretation. Reduced lactate levels (*p* < 0.05; Table 3) indicate a downregulation of metabolic activity, a common response under energetically restrictive conditions (Zhu et al. 2021; Cecchetto et al. 2022; Molinaro et al. 2022). Concurrently, elevated hepatic glycogen levels (*p* < 0.05; Figure 5) suggest a strategy to buffer dehydration and sustain basal metabolism, given glycogen's capacity to bind water (Zani et al. 2012; Holden et al. 2021; Yu et al. 2022). Together, these metabolic adjustments reinforce the view that seasonal stability in body mass masks substantial physiological restructuring. Nonetheless, the direct translation of such biochemical proxies into fitness outcomes remains complex and context‐dependent (Careau and Garland 2012; Dantzer et al. 2014; Dickens and Romero 2013; Mirante et al. 2024), highlighting the need for integrative, multilevel approaches to assess organismal performance (Costantini 2014). The seasonal physiological adjustments observed in female *L. arambarensis* support the hypothesis that energy allocation is tightly synchronized with reproductive demands rather than driven by metabolic dormancy. During winter, females preserved lipid and glycogen reserves while selectively catabolizing hepatic proteins, as evidenced by reduced liver protein and albumin levels. This pattern suggests reliance on protein‐derived gluconeogenesis to maintain glycemia while conserving lipids and carbohydrates as energetic and hydric buffers, a strategy reported for other lizards inhabiting seasonal environments (Cecchetto et al. 2019; Sánchez‐Loria et al. 2021; Oliveira et al. 2018). Importantly, these adjustments occurred without metabolic depression, corroborating previous evidence that *L. arambarensis* does not undergo brumation but instead remains physiologically active during colder months (Liz et al. 2019; A. Valgas et al. 2024), thereby rejecting the hypothesis of winter torpor.

In spring and summer, female physiology shifted toward reproductive investment, in agreement with the original prediction that vitellogenesis and egg production would dominate energy allocation. Increased gonadal development was accompanied by intensified feeding, elevated circulating metabolites associated with protein and lipid metabolism, and redistribution of substrates from liver and muscle tissues to the gonads. This reallocation culminated in summer, when muscular glycogen and lipid depletion reflected the high energetic costs of oviposition, mating, and foraging activities (Verrastro 2001; Oliveira et al. 2018; Ramírez‐Bautista et al. 2021). In autumn, following the reproductive peak, females redirected energy toward reserve replenishment, particularly glycogen and hepatic proteins, consistent with preparation for the subsequent winter and the next reproductive cycle. Together, these results support the hypothesis that females employ a flexible, seasonally modulated strategy that balances reproductive output with survival in a subtropical climate.

Male *L. arambarensis* exhibited comparatively stable body condition across seasons, supporting the assumption that sampled individuals belonged to a similar age cohort (Verrastro et al. 2003). Nevertheless, marked seasonal shifts in metabolic allocation were evident and aligned with predicted reproductive roles. During winter, males showed reduced surface activity and moderate feeding, accompanied by redistribution of circulating substrates into tissue glycogen and lipid reserves. This pattern suggests preparation for reproductive activities rather than metabolic suppression, consistent with reports of cold‐acclimatized lizards that maintain physiological activity while adjusting substrate use (Zani et al. 2012; Vega Parry et al. 2013; Cecchetto et al. 2022).

In spring, males increased feeding intensity and redirected energy toward liver and muscle tissues, particularly protein reserves, supporting energetically costly behaviors such as territorial defense and mate searching (Verrastro 2001; Fuxjager et al. 2022). Testicular metabolism indicated active spermatogenesis, confirming that males reach reproductive readiness earlier than females, as predicted. During summer, males relied more heavily on dietary intake than stored reserves, contrasting with females' substantial endogenous investment, and reflecting sex‐specific energetic trade‐offs typical of oviparous lizards (Pianka 2011). In autumn, males increased tissue glycogen and protein reserves while circulating metabolites declined, indicating preparation for winter survival. These findings support the hypothesis of reproductive synchrony between sexes, with males strategically allocating energy in advance of peak female reproductive investment.

Juveniles displayed a distinct energetic profile shaped primarily by growth and survival rather than reproduction, in line with the study's predictions. During winter, juveniles exhibited elevated blood glucose despite low feeding activity, suggesting reliance on hepatic gluconeogenesis and lipid catabolism. This response parallels cryoprotective mechanisms described for other *Liolaemus* species exposed to low temperatures (Cecchetto et al. 2019, 2022) and supports the hypothesis that juveniles employ endogenous substrates to buffer thermal and nutritional stress.

In spring, increased food availability enabled rapid somatic growth, reflected in higher body length and enhanced protein and lipid allocation to liver and muscle tissues, while glycogen reserves declined, likely fueling growth processes (Huey et al. 2021; Oliveira et al. 2018). Summer samples were dominated by recently hatched individuals, characterized by low carbohydrate reserves but elevated tissue lipids, consistent with yolk‐derived energy supporting early dispersal and exploratory behavior (Norval and Slater 2019; Wilson 2020). In autumn, juveniles showed pronounced depletion of energetic reserves and reduced stomach contents, indicating heightened vulnerability to food scarcity and intraspecific competition with adults (Pincheira‐Donoso 2012; A. Valgas et al. 2024). These patterns support the hypothesis that juvenile survival is the most seasonally constrained component of the population.

Contrasting sex and age classes reveals a coordinated but asymmetric energetic strategy within *L. arambarensis*. While adult males and females maintain stable body mass, their metabolic priorities diverge: females emphasize reserve conservation and reproductive output, whereas males allocate energy toward activity and reproductive behaviors. Juveniles, in contrast, prioritize growth and short‐term survival, exhibiting greater sensitivity to seasonal resource fluctuations. Notably, all groups remain physiologically active throughout winter, reinforcing the rejection of brumation as a life‐history trait in this species.

When viewed in a broader biogeographic context, *L. arambarensis* displays an intermediate strategy between tropical lizards with continuous reproductive cycles and temperate species with strong metabolic suppression. Similar to neotropical species, it exhibits reproductive seasonality coupled with high physiological plasticity; yet, like temperate taxa, it relies on strategic reserve management and winter biochemical adjustments (J. R. Warner 2014; Boretto et al. 2020; Pizarro et al. 2022). This combination underscores the importance of subtropical thermal variability in shaping energy allocation patterns.

## 5 Conclusion

This is the first study to elucidate the dynamics of intermediary metabolism and oxidative balance of *L. arambarensis* in natural environments over two seasonal cycles, capturing the adaptive ecophysiological dynamics of the species in response to abiotic dynamics generated by the seasons. It was possible to investigate the different metabolic strategies of male and female adults and juveniles of the species throughout the seasonal cycle, highlighting for females a significant energy investment in spring and especially in summer for reproduction, where they rely on hepatic and muscular tail energy reserves to invest in oocyte production and maturation. In males, we observed that their investment in gonadal maturation precedes that of females, with sperm production starting in autumn, peaking in winter and spring. In winter, they rely on tissue reserves to maintain this process, as indicated by empty stomach predominance, while in spring, this is mainly sustained by diet. For juveniles, the major energy investment is focused on somatic growth dynamics and survival. Each season appears to present a new homeostatic obstacle to survival. In summer, shortly after birth, juveniles must maintain high metabolism to search for food and invest in body growth. In autumn, they need to structure energy reserves to survive winter, competing for resources with adult animals. In winter, due to their lower body mass/volume ratio, they become more vulnerable to temperature changes, facing thermoregulatory challenges. In spring, they finally have surplus energy to invest in somatic growth, due to increased food availability. Throughout this study, we observed the tissue peculiarities of different age groups and sexes in ecological dynamics and energetic requirements for maintaining homeostasis. Studies aimed at understanding physiological adaptations of living beings in natural environments are increasingly important for conservation, as they provide information on the health status and fitness of the species, serving as predictive tools for survival in the face of environmental anthropogenic stressors such as climate change.

## Data Availability

The data that support the findings of this study are available in the supporting information of this article.

## References

[jez70091-bib-0002] Angilletta Jr., M. J. , and M. W. Sears . 2001. “The Metabolic Cost of Reproduction in an Oviparous Lizard.” Functional Ecology 15, no. 4: 404–413. 10.1046/j.1365-2435.2001.00534.x.

[jez70091-bib-0003] Araujo, J. F. , A. A. N. Valgas , D. R. de Oliveira , L. Verrastro , and G. T. Oliveira . 2024. “Pesticides Compromise Health: A Comparison Between Lizards Collected Within and Outside an Agricultural Area.” Environmental Monitoring and Assessment 196, no. 4: 334. 38430330 10.1007/s10661-024-12498-1

[jez70091-bib-0005] Beaulieu, M. , and D. Costantini . 2014. “Biomarkers of Oxidative Status: Missing Tools in Conservation Physiology.” Conservation Physiology 2, no. 1: cou014. 27293635 10.1093/conphys/cou014 PMC4806730

[jez70091-bib-0009] Boretto, J. M. , J. B. Fernández , F. Cabezas‐Cartes , M. S. Medina , and N. R. Ibargüengoytía . 2020. “Reproductive Biology of Lizards From Patagonia, Argentina: Physiological and Behavioral Adaptations to Cold and Harsh Environments.” In Lizards of Patagonia, 335–371. Springer.

[jez70091-bib-0014] Capdevila, P. , I. Stott , J. Cant , et al. 2022. “Life History Mediates the Trade‐Offs Among Different Components of Demographic Resilience.” Ecology Letters 25: 1566–1579. 35334148 10.1111/ele.14004 PMC9314072

[jez70091-bib-0015] Careau, V. , and T. Garland Jr. 2012. “Performance, Personality, and Energetics: Correlation, Causation, and Mechanism.” Physiological and Biochemical Zoology 85, no. 6: 543–571. 10.1086/666970. 23099454

[jez70091-bib-0016] Cecchetto, N. R. , S. M. Medina , F. Baudino , and N. R. Ibargüengoytía . 2022. “Wintertime Tales: How the Lizard *Liolaemus lineomaculatus* Endures the Temperate Cold Climate of Patagonia, Argentina.” Anais da Academia Brasileira de Ciências 94. 10.1590/0001-3765202220210758. 36228302

[jez70091-bib-0017] Cecchetto, N. R. , S. M. Medina , S. Taussig , and N. R. Ibargüengoytía . 2019. “The Lizard Abides: Cold Hardiness and Winter Refuges of *Liolaemus pictus argentinus* in Patagonia, Argentina.” Canadian Journal of Zoology 97, no. 9: 773–782.

[jez70091-bib-0020] Clavijo‐Baquet, S. , M. J. Orellana , P. Sabat , and F. Bozinovic . 2022. “How Do Ectotherms Perform in Cold Environments? Physiological and Life‐History Traits in an Andean Viviparous Lizard.” Frontiers in Ecology and Evolution 10: 974968.

[jez70091-bib-0021] Costantini, D. 2014. Oxidative Stress and Hormesis in Evolutionary Ecology and Physiology: A Marriage Between Mechanistic and Evolutionary Approaches, 362. Springer.

[jez70091-bib-0022] Costantini, D. 2022. “A Meta‐Analysis of Impacts of Immune Response and Infection on Oxidative Status in Vertebrates.” Conservation Physiology 10, no. 1: coac018. 35492421 10.1093/conphys/coac018 PMC9040321

[jez70091-bib-0023] Cox, N. , B. E. Young , P. Bowles , et al. 2022. “A Global Reptile Assessment Highlights Shared Conservation Needs of Tetrapods.” Nature 605, no. 7909: 285–290. 35477765 10.1038/s41586-022-04664-7 PMC9095493

[jez70091-bib-0025] Cubas, G. K. , S. F. Gohlke , T. Q. Vieira , and L. Verrastro . 2024. “Reduction in the Geographic Range of the Microendemic Sand Dune Lizard *Liolaemus arambarensis* Verrastro et al.” Herpetology Notes 17: 767–769.

[jez70091-bib-0026] Dantzer, B. , Q. E. Fletcher , R. Boonstra , and M. J. Sheriff . 2014. “Measures of Physiological Stress: A Transparent or Opaque Window into the Status, Management and Conservation of Species?” Conservation Physiology 2, no. 1: cou023. 27293644 10.1093/conphys/cou023 PMC4732472

[jez70091-bib-0027] Dayananda, B. , S. B. Bezeng , S. Karunarathna , and R. A. Jeffree . 2021. “Climate Change Impacts on Tropical Reptiles: Likely Effects and Future Research Needs Based on Sri Lankan Perspectives.” Frontiers in Ecology and Evolution 9: 688723.

[jez70091-bib-0030] Dickens, M. J. , and L. M. Romero . 2013. “A Consensus Endocrine Profile for Chronically Stressed Wild Animals Does Not Exist.” General and Comparative Endocrinology 191: 177–189. 23816765 10.1016/j.ygcen.2013.06.014

[jez70091-bib-0031] Domenici, P. , and F. Seebacher . 2020. “The Impacts of Climate Change on the Biomechanics of Animals.” Conservation Physiology 8, no. 1: coz102. 31976075 10.1093/conphys/coz102 PMC6956782

[jez70091-bib-0032] Folch, J. , M. Lees , and G. H. S. Stanley . 1957. “A Simple Method for the Isolation and Purification of Total Lipides From Animal Tissues.” Journal of Biological Chemistry 226: 497–509. 13428781

[jez70091-bib-0033] Frings, C. S. , and R. T. Dunn . 1970. “A Colorimetric Method for Determination of Total Serum Lipids Based on the Sulfo‐Phospho‐Vanillin Reaction.” American Journal of Clinical Pathology 53: 89–91. 5410040 10.1093/ajcp/53.1.89

[jez70091-bib-0034] Fuxjager, M. J. , L. Fusani , and B. A. Schlinger . 2022. “Physiological Innovation and the Evolutionary Elaboration of Courtship Behaviour.” Animal Behaviour 184: 185–195.

[jez70091-bib-0037] Giacometti, D. , A. V. Palaoro , L. C. Leal , and F. C. de Barros . 2024. “How Seasonality Influences the Thermal Biology of Lizards With Different Thermoregulatory Strategies: A Meta‐Analysis.” Biological Reviews 99, no. 2: 409–429. 37872698 10.1111/brv.13028

[jez70091-bib-0039] Gómez Alés, R. , J. C. Acosta , and A. Laspiur . 2017. “Thermal Biology in Two Syntopic Lizards, *Phymaturus extrilidus* and *Liolaemus parvus*, in the Puna Region of Argentina.” Journal of Thermal Biology 68: 73–82. 28689724 10.1016/j.jtherbio.2017.02.001

[jez70091-bib-0041] Gunderson, A. R. , and J. H. Stillman . 2014. “Plasticity in Thermal Tolerance Has Limited Potential to Buffer Ectotherms From Global Warming.” Proceedings of the Royal Society B: Biological Sciences 281, no. 1776: 20132675. 10.1098/rspb.2015.0401 PMC4455808 25994676

[jez70091-bib-0043] Hau, M. , M. F. Haussmann , T. J. Greives , et al. 2015. “Repeated Stressors in Adulthood Increase the Rate of Biological Ageing.” Frontiers in Zoology 12, no. 4: 4. 25705242 10.1186/s12983-015-0095-z PMC4336494

[jez70091-bib-0045] Holden, K. G. , E. J. Gangloff , E. Gomez‐Mancillas , K. Hagerty , and A. M. Bronikowski . 2021. “Surviving Winter: Physiological Regulation of Energy Balance in a Temperate Ectotherm Entering and Exiting Brumation.” General and Comparative Endocrinology 307: 113758. 33771532 10.1016/j.ygcen.2021.113758

[jez70091-bib-0048] Huey, R. B. , D. B. Miles , and E. R. Pianka . 2021. “Seasonality in Kgalagadi Lizards: Inferences From Legacy Data.” American Naturalist 198, no. 6: 759–771. 10.1086/716895 34762567

[jez70091-bib-0057] LeBas, N. R. , and N. J. Marshall . 2000. “The Role of Colour in Signalling and Male Choice in the Agamid Lizard *Ctenophorus ornatus* .” Proceedings of the Royal Society of London. Series B: Biological Sciences 267, no. 1442: 445–452. 10.1098/rspb.2000.1020 PMC1690562 10737400

[jez70091-bib-0058] Li, C. , S. Chen , L. Xia , et al. 2025. “Life‐History Traits Trade‐Off in Gecko (*Gekko japonicus*) Under the Influence of Climate Warming and Spirotetramat: Different Adaptations to Stressors in Female and Male.” Science of the Total Environment 958: 177978. 39657339 10.1016/j.scitotenv.2024.177978

[jez70091-bib-0060] Lincoln, G. 2019. “A Brief History of Circannual Time.” Journal of Neuroendocrinology 31, no. 3: e12694. 30739343 10.1111/jne.12694

[jez70091-bib-0062] Ling, Y. F. , and T. C. Bonebrake . 2022. “Consistent Heat Tolerance under Starvation Across Seasonal Morphs in *Mycalesis mineus* (Lepidoptera: Nymphalidae).” Comparative Biochemistry and Physiology. Part A, Molecular & Integrative Physiology 271: 111261. 10.1016/j.cbpa.2022.111261 35728756

[jez70091-bib-0063] Liz, A. V. , V. Santos , T. Ribeiro , M. Guimarães , and L. Verrastro . 2019. “Are Lizards Sensitive to Anomalous Seasonal Temperatures? Long‐Term Thermobiological Variability in a Subtropical Species.” PLoS One 14, no. 12. 10.1371/journal.pone.0226399. PMC6922334 31856183

[jez70091-bib-0064] Longhini, L. S. , L. S. Porto , A. Rocha , K. C. Bícego , W. Klein , and L. H. Gargaglioni . 2019. “Seasonal Variation of Hypoxic and Hypercarbic Ventilatory Responses in the Lizard *Tropidurus torquatus* .” Comparative Biochemistry and Physiology. Part A, Molecular & Integrative Physiology 237: 110534. 10.1016/j.cbpa.2019.110534 31401309

[jez70091-bib-0068] Mi, C. , L. Ma , Y. Wang , D. Wu , W. Du , and B. Sun . 2022. “Temperate and Tropical Lizards Are Vulnerable to Climate Warming Due to Increased Water Loss and Heat Stress.” Proceedings of the Royal Society B: Biological Sciences 289: 20221074. 10.1098/rspb.2022.1074. PMC9363995 35946157

[jez70091-bib-0070] Mirante, D. , L. Santini , D. Costantini , and A. Benítez‐López . 2025. “Glucocorticoid Responses of Wildlife to Anthropogenic Stressors Are Influenced by Disturbance Type and Species Traits.” Functional Ecology 39, no. 3: 681–697.

[jez70091-bib-0071] Molinaro, H. G. , G. S. Anderson , L. Gruny , E. S. Sperou , and D. J. Heard . 2022. “Use of Blood Lactate in Assessment of Manual Capture Techniques of Zoo‐Housed Crocodilians.” Animals: An Open Access Journal From MDPI 12, no. 3: 397. 35158720 10.3390/ani12030397 PMC8833426

[jez70091-bib-0074] Naya, D. E. , C. Veloso , and F. Bozinovic . 2008. “Physiological Flexibility in the Andean Lizard *Liolaemus bellii*: Seasonal Changes in Energy Acquisition, Storage and Expenditure.” Journal of Comparative Physiology B 178, no. 8: 1007–1015. 10.1007/s00360-008-0292-6 18626649

[jez70091-bib-0076] Norval, G. , and K. Slater . 2019. “Interrelation of Fat Body Mass, Liver Mass, and Environmental Parameters on the Reproductive Cycle of the Brown Anole (*Anolis sagrei*), an Introduced Lizard in Taiwan.” Herpetological Conservation and Biology 14: 67–79.

[jez70091-bib-0077] Oliveira, M. R. , F. M. Braghirolli , L. E. Krause Lanés , L. Verrastro , and G. T. Oliveira . 2021. “Evaluation of the Seasonal Variation of Parameters of Oxidative Status of *Tropidurus catalanensis* Gudynas and Skuk, 1983.” South American Journal of Herpetology 19, no. 1: 12–21.

[jez70091-bib-0078] Oliveira, M. R. , F. M. Braghirolli , L. Verrastro , and G. T. Oliveira . 2018. “Seasonal and Sexual Variation of the Intermediate Metabolism and Body Condition Indexes in the Lizard *Tropidurus catalanensis* (Gudynas and Skuk, 1983) (Squamata: Tropiduridae).” South American Journal of Herpetology 13, no. 1: 85–95.

[jez70091-bib-0081] Pereira, R. J. 2021. “Climate Change and Accelerated Aging in Ectotherms: A Framework for Understanding the Consequences of Increased Metabolic Rates and Oxidative Stress.” Global Change Biology 27, no. 24: 6360–6374.

[jez70091-bib-0082] Pianka, E. R. 2011. *Evolutionary Ecology*. Eric R. Pianka.

[jez70091-bib-0083] Pincheira‐Donoso, D. 2012. “Intraspecific Predation in the Liolaemus Lizard Radiation: A Primer.” Animal Biology 62, no. 3: 277–287.

[jez70091-bib-0084] Pizarro, J. E. , A. Laspiur , J. C. Acosta , G. M. Blanco , and J. M. Boretto . 2022. “High Reproductive Effort in a Vulnerable Lizard From High Altitudes in Argentina: Reproductive Biology and Sexual Dimorphism in *Phymaturus extrilidus* .” Supplement, Anais da Academia Brasileira de Ciências 94, no. S4: e20210179. 36515324 10.1590/0001-3765202220210179

[jez70091-bib-0086] Ramírez‐Bautista, A. , J. W. Sites Jr. , J. C. Marshall , et al. 2021. “Reproduction and Sexual Dimorphism in the Viviparous Lizard *Sceloporus palaciosi* (Squamata: Phrynosomatidae) From the Trans‐Mexican Volcanic Belt, Mexico.” Acta Zoologica 102, no. 1: 63–76.

[jez70091-bib-0090] Sánchez‐Loria, O. , E. Gomez , O. Arce , and S. Chamut . 2021. “Metabolic and Hormonal Changes Associated With Vitellogenesis in *Salvator merianae* Lizards.” Zoological Science 38, no. 5: 459–465. 34664921 10.2108/zs210013

[jez70091-bib-0092] Silveira, A. L. , C. F. D. da Rocha , C. Nogueira , et al. 2021. “*Liolaemus arambarensis*.” The IUCN Red List of Threatened Species 2021: e.T178744A159254568. 10.2305/IUCN.UK.2021-3.RLTS.T178744A159254568.pt.

[jez70091-bib-0093] Stellatelli, O. A. , A. Villalba , C. Block , L. E. Vega , J. E. Dajil , and F. B. Cruz . 2018. “Seasonal Shifts in the Thermal Biology of the Lizard *Liolaemus tandiliensis* (Squamata, Liolaemidae).” Journal of Thermal Biology 73: 61–70. 29549992 10.1016/j.jtherbio.2018.02.009

[jez70091-bib-0098] Valgas, A. , G. K. Cubas , D. R. de Oliveira , et al. 2024. “Ecophysiological Responses of *Liolaemus arambarensis* Juveniles to Experimental Temperature Variations.” Comparative Biochemistry and Physiology. Part A, Molecular & Integrative Physiology 290: 111577. 10.1016/j.cbpa.2024.111577 38228266

[jez70091-bib-0099] Valgas, A. A. N. , N. M. A. Wingen , S. H. D. Santos , G. T. Oliveira , and P. B. Araujo . 2020. “Biochemical‐Functional Parameters of Red Swamp Crayfish *Procambarus clarkii* (Girard, 1852) (Crustacea, Cambaridae) Female Throughout a Seasonal Cycle in Southeast Brazil.” Marine and Freshwater Behaviour and Physiology 53, no. 3: 113–129.

[jez70091-bib-0100] Van Handel, E. 1965. “Estimation of Glycogen in Small Amounts of Tissue.” Analytical Biochemistry 11: 256–265. 5840660 10.1016/0003-2697(65)90013-8

[jez70091-bib-0101] Vega Parry, H. , T. Alonso , H. Caldironi , and M. E. Manes . 2013. “Composition of Neutral Lipids and Phospholipids in Tegu Lizard *Tupinambis merianae* Fat Bodies.” Revista Argentina de Producción Animal 33, no. 2: 129–137.

[jez70091-bib-0102] Verrastro, L. 2001. “Descrição, Estratégia Reprodutiva e Alimentar De Uma Nova Espécie Do Gênero Liolaemus No Rio Grande Do Sul, Brasil (Iguania: Tropiduridae).” Doctoral diss., Universidade Federal de São Carlos.

[jez70091-bib-0103] Verrastro, L. , L. Veronese , C. Bujes , and M. M. Dias Filho . 2003. “A New Species of Liolaemus From Southern Brazil (Iguania: Tropiduridae).” Herpetologica 59, no. 1: 105–118.

[jez70091-bib-0106] Wang, Y. , G. Zhang , H. Jiang , D. Liu , X. Hu , and F. Qian . 2022. “Dynamic Changes in the Hormones of Black‐Necked Cranes During Reproduction.” Conservation Physiology 10, no. 1: coac040. 36937702 10.1093/conphys/coac040 PMC10020981

[jez70091-bib-0109] Warner, J. R. 2014. “Comparative Cold‐Hardiness Capacities of South American Lizards in the Genus *Liolaemus*.” Doctoral diss. California State University, Northridge).

[jez70091-bib-0112] Wikelski, M. , and S. J. Cooke . 2006. “Conservation Physiology.” Trends in Ecology & Evolution 21, no. 1: 38–46. 16701468 10.1016/j.tree.2005.10.018

[jez70091-bib-0113] Wikelski, M. , and C. Thom . 2000. “Marine Iguanas Shrink to Survive El Niño.” Nature 403, no. 6765: 37–38. 10638740 10.1038/47396

[jez70091-bib-0114] Wilson, R. C. 2020. “Energy Stores and Life‐History Transitions in Red‐Sided Garter Snakes (*Thamnophis sirtalis parietalis*).” Doctoral diss., Portland State University.

[jez70091-bib-0115] Yu, S. , Z. Wang , L. Zhang , et al. 2022. “Possible Changes in Trade‐Off Strategy in Female Lizards (*Eremias argus*) During Hibernation Following Exposure to Chlorantraniliprole: Impact on the HPG Axis and Energy Mobilization.” Pesticide Biochemistry and Physiology: 105059. 10.1016/j.pestbp.2022.105059. 35715026

[jez70091-bib-0116] Zani, P. A. , J. T. Irwin , M. E. Rollyson , et al. 2012. “Glycogen, Not Dehydration or Lipids, Limits Winter Survival of Side‐Blotched Lizards (*Uta stansburiana*).” Journal of Experimental Biology 215, no. 17: 3126–3134. 22875774 10.1242/jeb.069617

[jez70091-bib-0119] Zhu, X. , X. Qiu , X. Tang , and Y. Qi . 2021. “Tail Display Is Regulated by Anaerobic Metabolism in an Asian Agamid Lizard.” Integrative Zoology 16, no. 5: 729–740. 33733614 10.1111/1749-4877.12536

